# Early-childhood temperament moderates the prospective associations of coping with adolescent internalizing and externalizing symptoms

**DOI:** 10.3389/fpsyg.2022.1011095

**Published:** 2022-11-10

**Authors:** Michele R. Smith, Krystal H. Parrish, Lisa Shimomaeda, Maureen Zalewski, Maya L. Rosen, Alexandra Rodman, Steven Kasparek, Makeda Mayes, Andrew N. Meltzoff, Katie A. McLaughlin, Liliana J. Lengua

**Affiliations:** ^1^Department of Psychology, University of Washington, Seattle, WA, United States; ^2^Department of Psychology, University of Oregon, Eugene, OR, United States; ^3^Smith College, Northampton, MA, United States; ^4^Department of Psychology, Harvard University, Cambridge, MA, United States

**Keywords:** temperament, coping, adolescence, internalizing and externalizing behavior, COVID-19, early childhood, stress

## Abstract

While appraisal and coping are known to impact adolescent psychopathology, more vulnerable or resilient responses to stress may depend on individual temperament. This study examined early life temperament as a moderator of the prospective relations of pre-adolescent appraisal and coping with adolescent psychopathology. The sample included 226 (62% female, 14–15 years) adolescents with assessments starting at 3 years of age. Adolescents were predominately White (12% Black 9% Asian, 11% Latinx, 4% Multiracial, and 65% White). Observed early-childhood temperament (fear, frustration, executive control, and delay ability) were tested as moderators of pre-adolescent coping (active and avoidant) and appraisal (threat, positive) on internalizing and externalizing symptoms during the pandemic. Interaction effects were tested using regression in *R*. Sex and family context of stress were covariates. Early-childhood temperament was correlated with pre-adolescent symptoms, however, pre-adolescent appraisal and coping but not temperament predicted adolescent psychopathology. Frustration moderated the relations of active and avoidant coping and positive appraisal to symptoms such that coping and appraisal related to lower symptoms only for those low in frustration. Executive control moderated the associations of avoidant coping with symptoms such that avoidance reduced the likelihood of symptoms for youth low in executive control. Findings underscore the role of emotionality and self-regulation in youth adjustment, with the impact of coping differing with temperament. These findings suggest that equipping youth with a flexible assortment of coping skills may serve to reduce negative mental health outcomes.

## Introduction

**Figure 1:**
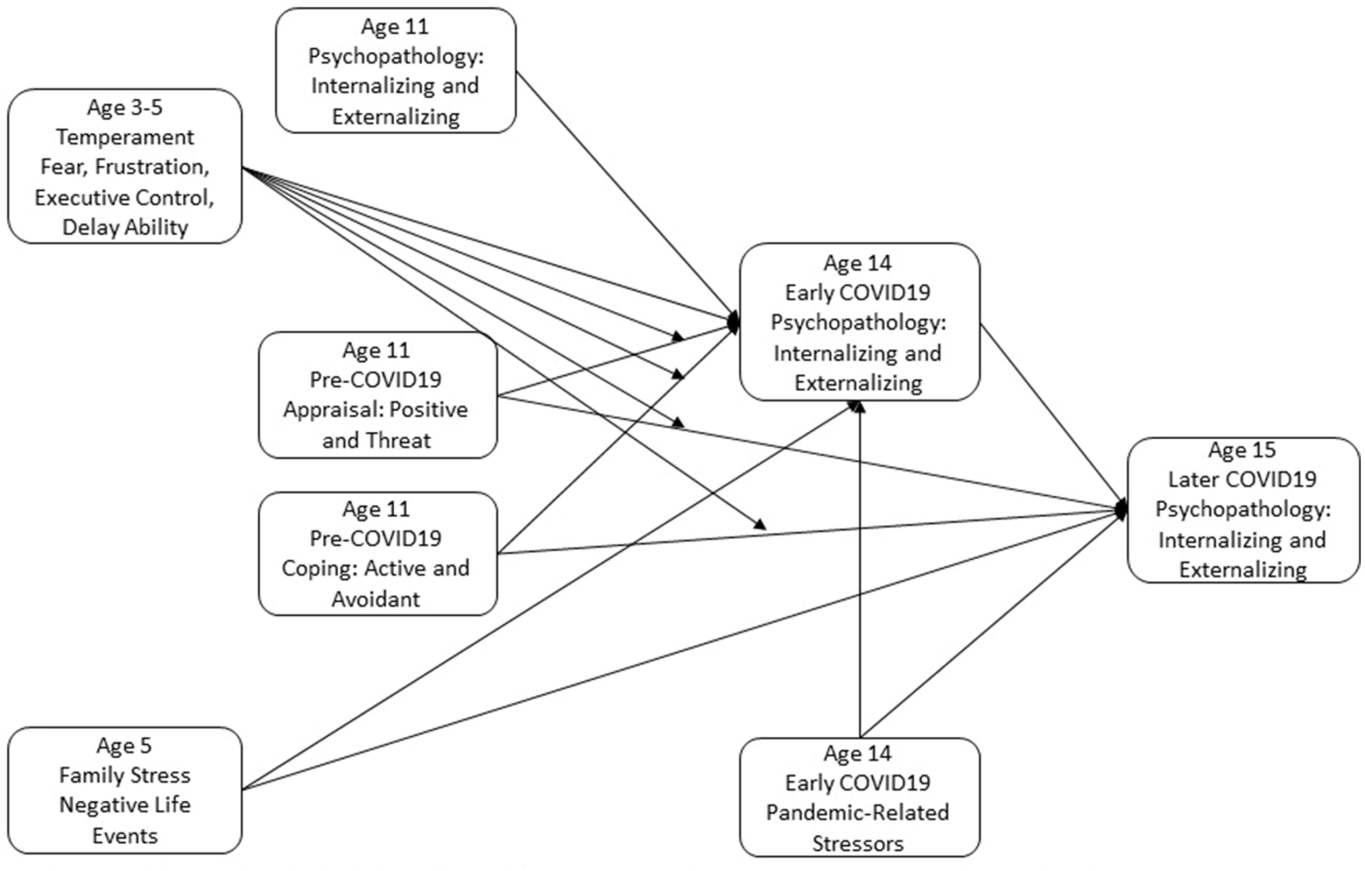
Conceptual model of early childhood temperament moderating the associations of appraisal and coping styles with levels of and changes in psychopathology in response to the context of stress posed by the COVID19 pandemic.

Adolescence is a time when youth experience increases in psychopathology (Kessler et al., 2009; Costello et al., 2011), and early onset of psychopathology is associated with a more persistent course (Moffitt et al., 2007; Costello et al., 2016). For youth who have experienced adverse life events, rates of psychopathology are 2–4 times those of other youth (McLaughlin et al., 2012). Temperament (Nigg, 2006; Rothbart and Posner, 2006) and appraisal and coping (Compas et al., 2017), that is, the assessment and effortful management of a stressor, have also been shown to contribute to youth psychopathology above the effects of experiences of stress or adversity (Wadsworth and Berger, 2006; Pitzer et al., 2011; Rabinowitz et al., 2016; Chung et al., 2019). In fact, the effectiveness of appraisal and coping behaviors in reducing psychopathology may vary based on individual differences in temperament. This study examined how temperament might alter the effects of youth coping with stress on their mental health symptoms. This combination of characterological and intentional emotion regulation efforts was expected to predict adolescent psychopathology in response to stress. We examined early-childhood temperament as a moderator of the prospective effects of pre-adolescent appraisal and coping on adolescent psychopathology while accounting for past and concurrent contexts of adversity and stress (see Figure 1).

Temperament is a consistent and robust predictor of psychopathology (e.g., Nigg, 2006) and may operate through its interactions with other risk factors (e.g., Rothbart and Bates, 2006). Temperament is conceptualized as biologically based individual differences in patterns of reactivity and self-regulation that are relatively stable over time but may be influenced by experience (Rothbart and Bates, 2006; Rothbart, 2007). There are multiple facets of temperament. Fear reactivity (negative emotion related to anticipation of threat or distress) and frustration (negative emotion regarding goal blocking or interruption of goals or tasks) are prominent facets of temperament negative emotionality (Rothbart, 2007). Fear reactivity arises from activation of the behavioral inhibition system, associated with responsiveness to cues of threat or punishment and freezing or passive avoidance responses, while frustration reactivity arises from initiation of the behavioral activation system which is associated with responsiveness to reward cues, frustration in non-reward contexts, active avoidance of punishment, as well as the fight-flight system responsible for defensive aggression (McNaughton and Gray, 2000; Rothbart et al., 2014). Effortful control, comprised both executive control and delay ability, refers to individual differences in executive regulation of attention and inhibitory control of thoughts and behaviors (Rothbart and Bates, 2006). Executive Control (EC) is the non-emotional cognitive component that involves shifting and focusing attention and the inhibition and activation of behavior, whereas Delay Ability (DA) refers to the motivational component that involves delaying an immediate reward for a larger reward later (Rothbart et al., 2000; Rothbart and Bates, 2006; Kim et al., 2013).

Both negative emotionality and effortful control have been associated with internalizing and externalizing problems in youth (e.g., Eisenberg et al., 2005; Lengua, 2006). Indeed, youth high in negative emotionality are at risk for both internalizing and externalizing problems (e.g., Eisenberg et al., 2005). Independently, fear and frustration have been related to adjustment. Fear is a consistent predictor of higher internalizing, while frustration has been associated with both internalizing and externalizing problems (see De Pauw and Mervielde, 2010 for review; Rothbart, 2007). Executive control is consistently associated with both positive and negative indicators of adjustment, including internalizing and externalizing problems (Lengua et al., 2015; Kim-Spoon et al., 2019), while lower delay ability has been related increased externalizing symptoms in children (Gusdorf et al., 2011; Lengua et al., 2015). Little evidence supports a connection between delay ability and internalizing symptoms. However, one study suggested that low reward sensitivity (a facet of delay inability) was related to increase internalizing psychopathology in adolescents (Forbes et al., 2017).

The vulnerability model of temperament, however, suggests that particular temperament profiles may be associated with poor adjustment through their interaction with other factors (Nigg, 2006). In a review of the role of temperament in adolescent psychopathology, a vulnerability model emerged most consistently (Tackett, 2006). This diathesis-stress approach suggests that temperament may create risk or resilience to psychopathology under high or low risk conditions. That is, temperament may moderate environmental risk or behavior to influence adjustment (Ingram and Luxton, 2005; Nigg, 2006; Ingram and Price, 2010). Indeed, there is considerable evidence to suggest this process (Roisman et al., 2012; Rioux et al., 2016). In addition to temperament, appraisal and coping styles may be critical factors in youth responses to stressors, but their effectiveness might depend on temperament.

Appraisal and coping reflect cognitive approach and volitional regulation processes regarding individual perception and response to a stressor (Folkman, 1984; Compas et al., 2001). Appraisals refer to the assessment of an event as stressful or not, and whether one has the resources to deal with the stressful event. Appraisals can be characterized as positive and threat appraisals. Positive appraisals include challenge (evaluation of the potential for gain or positive outcomes) and resource (evaluation that one has the resources to deal with the event) appraisals. Threat appraisals, on the other hand, are an assessment of harm or future loss. Positive appraisals have been related to fewer adjustment problems whereas threat appraisals have been associated with greater adjustment problems (Sheets et al., 1996; Lengua et al., 1999; Jackson and Warren, 2000; Lengua and Long, 2002; Raver et al., 2016).

Coping traditionally describes specific, volitional, and intentional self-regulatory strategies employed when faced with stress that has been appraised as exceeding one’s resources (Compas et al., 2001, 2017). Coping is commonly operationalized as active or avoidant. Active coping strategies involve directing oneself towards/dealing with the problem or related emotions, whereas avoidant coping strategies involve removing oneself/withdrawing from the stressful situation and associated emotions. A large body of research has examined processes of dealing with stress in youth and has identified specific coping strategies that are differentially associated with emotional and behavioral adjustment (Compas et al., 2017).

As children age, temperament may aid or hinder propitious appraisal and coping. In their theoretical differential-choice effectiveness model, Bolger and Zuckerman (1995) found that individual personality may be related to differences in the effectiveness of coping strategies on psychopathology. A small body of more recent research has supported this phenomenon in youth (Blair et al., 2004; Muris, 2006; Miller et al., 2009). For example, using parent-reported measures, researchers found that active coping strategies moderated the association between negative emotionality and internalizing symptoms in a sample of youth with cancer (Miller et al., 2009). In another study, children’s self-regulation, assessed as approach-flexibility, moderated coping, such that at higher levels of self-regulation, active coping was related to lower anxiety and avoidant coping was unrelated to anxiety (Lengua and Sandler, 1996). Such interaction effects indicate that, over and above direct effects of temperament, appraisal, and coping on psychopathology, their interactions are relevant. In particular, higher negative emotionality and lower effortful control might render active coping efforts less effective and might exacerbate the negative effects of avoidant coping.

These effects might be even more pronounced as youth have navigated the substantial stress and disruptions associated with the COVID-19 pandemic. The pandemic has been largely associated with increased adjustment problems in youth (Barendse et al., 2021; Samji et al., 2021; Chadi et al., 2022). In particular, avoidant coping before and during the pandemic was related to worse mental health outcomes (Liang et al., 2020; Tyra et al., 2021), while active coping strategies were related to better outcomes (Arbel et al., 2020). Moreover, in studies prior to the pandemic, temperament, appraisal, and coping were shown to contribute to youth psychopathology above the effects of experiences of stress or adversity (Pitzer et al., 2011; Rabinowitz et al., 2016). Given that appraisal and coping styles are moderately stable in preadolescence and adolescence (e.g., Thompson et al., 2016), they may be relevant prospective predictors of how youth respond to stressors such as those related to the pandemic.

It is essential to understand how temperament, appraisal, and coping styles inform adolescent adjustment in contexts of stress and adversity. This study is unique in prospectively testing the combined effects of temperament, appraisal, and coping on psychopathology across developmental periods. We examined early-childhood temperament as a moderator of the prospective effects of appraisal and coping styles on levels and changes in youth adjustment during the COVID-19 pandemic, while accounting for the context of stress both before and during the pandemic. We expected that appraisal and coping styles prior to the pandemic would predict levels of psychopathology early in the pandemic, as well as changes across 6 months of the pandemic. However, we expected the associations of prospective associations of appraisal and coping with youth adjustment during the pandemic would be dependent on temperament. Specifically, high fear and frustration and low effortful control (composed of executive control and delay ability) were hypothesized as risk factors, increasing the negative impact of threat appraisal or avoidant coping on psychopathology, while reducing the positive impact of positive appraisal and active coping.

## Materials and methods

### Participants

The study used a sample of adolescents from a larger community-based sample of 306 children and their mothers who were assessed at multiple time-points across childhood. The subset of 226 participants participated in an age-12 assessment, capturing adjustment before the pandemic. Participants from the parent study were excluded from age-12 assessments based on the following criteria: moved out-of-area, IQ < 80, active substance dependence, psychosis, or the presence of pervasive developmental disorder. All subjects who participated in the age-12 assessment were invited to complete COVID-19 surveys. Of those, 143 adolescents (63%, 62 female, mean age = 14.33, *SD* = 0.48) and a caregiver completed online questionnaires between April and May of 2020, early in the COVID-19 pandemic (spring 2020), and 152 youth (67%, 72 female, mean age 14.87, SD = 0.49), and a caregiver completed questionnaires online between November 2020 and January 2021 (winter 2020–21). Some participants completing the second survey had not completed the first one, and vice versa, resulting in a total of 161 survey respondents across the two surveys. Of those participants, 105 (65%) identified as White, 19 (12%) as Black, 17 (11%) as Latinx, 14 (9%) as Asian, and 6 (4%) as another race or ethnicity.

### Missingness analyses

Participants completing both the spring 2020 and winter 2020–21 COVID-19 surveys were compared with those who did not complete either of the surveys. We compared variables across participants missing and not missing COVID-19 survey by examining the magnitude and significance of point biserial correlations with missingness coded as 0 for no missing data, and 1 for missing either COVID-19 survey. Families who did not complete the COVID-19 surveys did not differ significantly from the families who completed the survey on T1-4 temperament variables, T4 negative life events, T5 (age-12) income-to-needs, appraisal, and coping or child internalizing or externalizing problems. The magnitude of missingness effects were small (*r* = |0.009–0.129|), indicating that it was unlikely that missing data introduced bias in the model estimates, and missing data would likely have minimal impact on parameter estimates (Collins et al., 2001; Dong and Peng, 2013). Full-information maximum likelihood estimation (FIMLE) was considered appropriate under these conditions and consistently produces less biased parameter estimates and greater statistical power (e.g., Enders and Bandalos, 2001).

### Procedure

This study is part of a longitudinal study examining the development of self-regulation, in the context of early-childhood experiences of low income and its associated adversity. Parents and children granted consent and assent in advance of data collection. For Time 1–Time 4 assessments, mothers and children completed tasks and questionnaires in a university lab setting. Families received compensation at each visit. Beginning when the youth were roughly 36-month old, the first four time-points were separated by 9 months (T1 child age *M* = 3.06 years, *SD* = 0.07, T4 child age *M* = 5.35 years, *SD* = 0.28). The fifth time-point (T5) was approximately 6 years after T4 when youth were age 10–13 years (*M* = 11.00, *SD* = 0.59), and approximately 3 years later at T6, (M = 14.33, *SD* = 0.48) COVID-19 experiences and adjustment symptoms were assessed using youth and parent report on an online survey conducted in April/May 2020 coinciding with stay home orders. Identical surveys were administered again in November 2020–January 2021 (T7, *M* = 14.87, *SD* = 0.49). The university Institutional Review Board approved all procedures for this study.

### Measures

#### Demographics

At Time 1, mothers reported demographic characteristics including family income and child sex. Mothers reported on household income from all sources on a 14-point Likert scale that provided a fine-grained breakdown of income at the lower levels facilitating identification of families at the federal poverty cutoff using an income-to-needs ratio (e.g., *1* = $14,570 or less, *2* = $14,571–$18,310, *3* = $18,311–22,050, etc.). Families were recruited into the original study to equally represent the full range of income, and as a result, family income and the income-to-needs ratio were highly correlated (*r* = 0.92). Therefore, the 14-point variable representing the full range of income was used for analyses [*M* = 8.75 (≈$38–$39 K), *SD* = 3.93, *Range* = 1.00 ($14,570 or less)–14.00 (above $150 K)]. Correlations among T1–T4 income ranged from 0.80 to 0.88. Given the high stability in income, only T4 income was analyzed.

#### T4 negative life events

Mother-report on the General Life Events Schedule for Children (Sandler et al., 1986) assessed negative life events. Mothers reported whether the 28 moderate-to-major negative life events occurred during the previous year, and the total score was the summed number of events.

#### T1–T4 temperament

Temperament was assessed with behavioral observations at the first four timepoints when children were 3–5 years old. For this study, temperament measures were the average of task scores across the four timepoints. Observed measures of children’s fear and frustration were adapted from the Laboratory Temperament Assessment Battery: Preschool Version (Goldsmith et al., 1993).

##### Fear

Fear reactivity was measured by the child’s response to a toy spider. After a toy spider was presented, the child received three cues to touch it. Fear was assessed on the intensity (0–2, no response to strong response) of behaviors by (1) how long it took to touch the spider, (2) physical response, (3) facial response, and (4) verbal response; scores were aggregated across behavior to comprise a fear score for each cue. Total fear reactivity score was calculated based on an average across the three cues, ICC = 0.78–0.97.

##### Frustration

The Transparent Box task assessed child frustration. In this task, children were faced with a toy locked inside a clear, impenetrable box. Children received the keys to the box and were asked to remove the toy; however, these were the wrong keys and did not open the box. For a 2-min period, the child worked alone to open the box. Frustration was assessed though the intensity (0–2; no response to strong response) of physical, facial, and verbal response, alongside expressed annoyance with the research assistant. The task was coded in 30-s intervals, and intervals were averaged to create a total frustration score, ICC = 0.72–0.79.

##### Executive control

Executive control was assessed as a composite of six tasks. The *NEPSY Inhibition* subtest assesses a child’s ability to inhibit a dominant response in order to enact a novel response. The *NEPSY Auditory Attention* subtest is a continuous performance test that assesses the ability to be vigilant and to maintain and shift selective auditory set. Total scores for both scales were the proportion of correct responses across the task.

Behavioral inhibitory control was assessed using the *Bear-Dragon task* (Kochanska et al., 1996; Li-Grining, 2007), which requires the child to perform actions when a directive is given by a bear puppet, but not when given by a dragon puppet. Children’s actions were scored as performing no movement, a wrong movement, a partial movement, or a complete movement, with scores ranging from 0 to 3. Total scores were the proportion of the score across both bear and dragon items to the total possible score.

Cognitive inhibitory control was assessed using the *Day-Night task* (Gerstadt et al., 1994), which requires the child to say “day” when shown a picture of moon and stars and “night” when shown a picture of the sun. Children’s actions were scored 1 for correctly providing the non-dominant response or 0 for providing the dominant response. Total scores were the proportion of correct responses.

The *Dimensional Change Card Sort* (Zelazo et al., 2003) assesses cognitive inhibitory control, attention focusing, and set shifting. In this task, children were introduced to two black recipe boxes with slots cut in the top. Target cards were attached to the front of each box. The target cards consisted of a silhouetted figure on a colored background (star on blue background and truck on red background). Children were instructed to sort cards according to either the shape or color properties on the target cards, first according to shape (six trials), then according to color (six trials). The experimenter stated the sorting rule before each trial, and then presented a card and labeled it according to the current dimension (e.g., on a shape trial, “Here’s a truck. Where does it go?”). If children correctly sorted >50% of cards, they advanced to the next level in which the target cards integrated the sorting properties. Target cards consisted of a colored figure on a white background (blue star and red truck), and children were again instructed to sort according to shape (six trials) and then color (six trials). If they correctly sorted >50% of the cards, children advanced to the next level in which they were instructed to sort by one dimension (color) if the card had a border on it and by the other dimension (shape) if the card lacked the border (12 trials). The score was the proportion of correct trials out of the total possible of 36 trials.

The *Head, Toes, Knees, Shoulders* (HTKS) task also integrates attention regulation and inhibitory control (Ponitz et al., 2008). Children are asked to follow the instructions of the experimenter, but to enact the opposite of what the experimenter directs (e.g., touch toes when asked to touch head). Behaviors were coded as 0 points if the child touched the directed body part, 1 point if the child self-corrected his/her behavior, and 2 points if the child only touched the opposite body part. Total scores were the proportion of the score across items to the total possible score. Twenty percent of all executive control tasks were independently re-scored to assess inter-rater reliability. ICCs on individual tasks ranged from 0.72 to 0.98. Consistent with previous research, an overall executive control score was computed as the mean of the proportion scores of the individual tasks. Internal consistency of the composite executive control measure was *α* = 0.67, and the ICC for the composite was 0.83, α = 0.67–0.74.

##### Delay ability

The ability to delay gratification was assessed using the gift delay task (Kochanska et al., 1996). During the gift delay task, the child was told that s/he would receive a present, but that the experimenter wanted to wrap it. The child was instructed to sit facing the opposite direction and to not peek while the experimenter noisily wrapped the gift. Children’s peeking behavior (frequency, degree, latency to peek, and latency to turn around) and difficulty with the delay (fidgeting, tensing, getting out of seat, grimacing, and talking) were rated. Latencies and behavior scores were converted to proportions of total possible times/scores and averaged, with higher delay scores reflecting greater ability to delay gratification. An overall delay ability score was computed as the mean of the proportion scores for the individual delay indicators. Internal consistency of the composite delay ability measure was *α* = 0.71–0.77, and the ICC was.91.

#### T5 threat and positive appraisal

**A**ppraisal styles were assessed using youth responses on the What I Felt Scale (Sheets et al., 1996), in which they were prompted to think about three of the “biggest problems” they had during the past month and rate on a Likert-type scale from “0 = not at all” to “3 = most of the time” how much they tended to think each of the thoughts related to those problems or problems like those. *Threat appraisal* included six dimensions of negative thoughts about life events: negative self-evaluations, negative evaluation by others, rejection, criticism of others, harm to others, and loss of desired objects or activities. *Positive appraisal* was assessed by combining the challenge appraisal subscale (seven items, e.g., “You thought that you would be able to figure the problem out”) and the resource appraisal subscale (six items, e.g., “You thought about all the people and things in your life that could help with the situation”). The threat and positive appraisal scales had good internal consistency α = 0.83–0.88 and.83–0.89, respectively.

#### T5 active and avoidant coping

Using the Children’s Coping Strategies Checklist (Ayers et al., 1996), youth rated (0, not at all to 3, most of the time) how often they used each coping behavior when they had problems during the previous month. They were prompted to think about problems like the ones identified for the appraisal measure above. *Active coping* included various strategies: cognitive decision making, control, direct problem solving, positive cognitive restructuring, optimism, and seeking understanding strategies. *Avoidant coping* included the strategies: cognitive avoidance, avoidant actions, and wishful thinking. The active and avoidant coping scales had good internal consistency α = 0.88–0.93 and.76–0.86, respectively.

#### T5, T6, and T7 psychopathology

Both mother and youth reported on psychopathology and combined to create cross-reporter measures of adjustment at T5, T6, and T7. Multi-method measures of adjustment were sought to partially address the effects of shared method variance and reporter bias on the observed associations. Relying on only one method of assessment for a construct can lead to ambiguous interpretation of the validity of a measure (Marsh and Grayson, 1995), and combining reporters has been suggested to capture differing perspectives of adjustment (e.g., Hinshaw and Park, 1999). At T5, pre-adolescent psychopathology was assessed by youth report on the Youth Self-Report (YSR) and parent report on the Child Behavior Checklist (CBCL; Achenbach, 1991; Achenbach et al., 2003). At the T6 and T7 assessments adolescents and parents completed the 25-item Strengths and Difficulties Questionnaire (SDQ; Goodman, 2001), selected to reduce participant burden, as it has substantially fewer items than the YSR and CBCL. The SDQ has good reliability and validity (Dickey and Blumberg, 2004; Goodman et al., 2010) and correlates strongly with the CBCL/YSR (Goodman and Scott, 1999).

### Analytic plan

All analyses were conducted in *R* 4.1.3 (R Core Team, 2022). Descriptive statistics and correlations between variables were estimated for each sample. We tested our hypotheses using *R*’s *lavaan* package, version 0.6–11 (Rosseel, 2012) with FIML estimation to account for missing data. We examined a series of two-step nested multivariate multiple linear regression models to examine the contributions of early-childhood temperament (T1–T4), coping and appraisal (T5), and their interactions in predicting adolescent symptoms of psychopathology early in the pandemic (T6), indicating changes from earlier levels of psychopathology likely related to the initial stressors introduced by the pandemic. We also examined temperament, appraisal, and coping styles as predictors of psychopathology several months after the start of the pandemic (T7) to assess the extent to which temperament, appraisal, and coping styles contributed to adolescent psychopathology in response to the persistent stress of the pandemic. These effects were tested as contributing to changes in youth psychopathology above the effects of early negative life events (T4) and concurrent pandemic-specific stressors (T6). Sex, early-childhood family income (all T4), and pre-adolescent symptoms (T5) were also included as covariates. To test for main effects, the first step of each model included internalizing and externalizing symptoms jointly regressed onto each facet of temperament, one coping or appraisal style variable (active coping, avoidant coping, positive appraisal, and threat appraisal), and covariates to better account for shared variance across outcomes and permit more direct comparisons of coefficients (Tabachnick and Fidell, 2013). Next, we added interactions between temperament and coping/appraisal in the second step to test interaction effects. Predictors were mean centered prior to multiplication to avoid nonessential multicollinearity (Cohen et al., 2013). Significant interactions were probed at 1 and 2 SDs above/below and at the mean of temperament consistent with procedures outlined by Aiken and West (1991).

## Results

### T6 pandemic-related stressors

Parent and youth reported on pandemic-related stressors including, financial, health, school, social, and physical environment stressors that occurred within the month prior to the first COVID-19 assessment (Weissman et al., 2021). Seven of the stressors were related to the health of participants or close others (e.g., contracting COVID-19); four were related to financial impacts of COVID-19 (e.g., parent lost a job); four were related to disruptions to social life related to social distancing, remote school, and suspended activities; and three related to household noise and crowding. Scores were the count of risk factors endorsed. Adolescent and parent reports were correlated *r* = 0.59 and were averaged to capture both perspectives. Scores ranged from 0 to 18. Although not included in this study, pandemic-related stressors were also measured at T7, with T6 and T7 measures correlated.50. *p* < 0.001, suggesting moderate stability of stressors during the pandemic.

### Descriptive statistics and correlations

We present descriptive statistics in Table 1. Overall, levels of internalizing (*M_T6_* = 4.19; M_T7_ = 4.99) and externalizing (*M_T6_* = 5.03; M_T7_ = 5.40) were slightly elevated based on published norms (youthinmind.com/SDQ norms).

**Table 1:** Descriptive statistics for sample demographics and all study variables.

Sex	62% Female		
Race/ethnicity	Asian American		9%
	Black		12%
	Latinx		11%
	White		65%
	Multiracial or otherwise defined		4%
		**M (SD)**	
Child Age	*T4*	5.35(0.28)	
	*T5*	11.00 (0.59)	
	*T6*	14.33 (0.48)	
	*T7*	14.87 (0.49)	
T4 Income		9.31 (3.83)	
T4 Negative Life Events		5.22 (2.78)	
T6 Pandemic-related Stressors		2.32 (1.75)	
T1-4 Fear		0.41 (0.23)	
T1-4 Frustration		0.24 (0.09)	
T1-4 Executive Control		0.56 (0.14)	
T1-4 Delay		0.73 (0.17)	
T5 Threat Appraisal		5.76 (5.86)	
T5 Positive Appraisal		18.63 (6.89)	
T5 Active Coping		31.92 (12.99)	
T5 Avoidant Coping		14.28 (6.66)	
T5 Internalizing		9.28 (5.88)	
T5 Externalizing		7.05 (4.59)	
T6 Internalizing		4.19 (2.89)	
T6 Externalizing		5.03 (2.74)	
T7 Internalizing		4.99 (3.30)	
T7 Externalizing		5.40 (3.11)	

M and SD are used to represent mean and standard deviation, respectively.

Income was negatively correlated with age 11/12 psychopathology (T5) but not with levels of psychopathology during the pandemic (T6 or T7). Income was positively correlated with early-childhood executive control and delay ability, and negatively correlated with frustration (T1–T4). Early-childhood executive control and delay ability were significantly correlated with age 11/12 psychopathology (T5), but generally not with psychopathology during the pandemic (T6 or T7), suggesting that early-childhood effortful control was related to level of psychopathology but not their changes in response to pandemic-related stressors. Early-childhood fear was related to higher internalizing at the start of the pandemic (T6), and frustration was correlated with T5 and T6 externalizing. Psychopathology during the pandemic (T6 and T7) had moderate, positive correlations with pandemic-related stressors, while correlations with pre-adolescent negative life events were smaller and less consistent. Positive appraisal and active coping at age 11/12 (T5) were negatively correlated with both internalizing and externalizing psychopathology during the pandemic at T7. Correlations are presented in Table 2.

**Table 2:** Correlations among study variables.

Variable	1	2	3	4	5	6	7	8	9	10	11	12	13	14	15	16	17
1. Sex																	
2. Income	−0.05																
3. Negative Life Events	−0.07	−0.09															
4. Pandemic-related Stressors	−0.09	−0.03	0.08														
5. Fear	−0.08	−0.10	−0.04	0.03													
6. Frustration	0.15^*^	−0.13^*^	−0.00	0.06	0.09												
7. Executive Control	−0.14^*^	0.32^**^	0.05	−0.09	−0.14^*^	−0.15^*^											
8. Delay	−0.22^**^	0.18^**^	−0.01	−0.03	−0.08	−0.26^**^	0.43^**^										
9. Threat Appraisal	0.03	−0.02	−0.02	0.05	0.19^**^	0.09	−0.03	−0.08									
10. Positive Appraisal	−0.10	0.08	−0.01	−0.04	−0.13	−0.11	−0.00	0.07	−0.32^**^								
11. Active Coping	−0.10	0.07	−0.05	−0.10	−0.16^*^	−0.09	−0.02	0.06	−0.23^**^	0.77^**^							
12. Avoidant Coping	−0.02	−0.06	0.02	−0.06	−0.06	0.01	−0.12	−0.05	0.20^**^	0.26^**^	0.44^**^						
13. T5 Internalizing	0.04	−0.21^**^	0.18^**^	0.08	0.04	0.09	−0.15^*^	−0.07	0.27^**^	−0.26^**^	−0.18^*^	0.21^**^					
14. T5 Externalizing	0.19^**^	−0.34^**^	0.31^**^	0.11	0.03	0.21^**^	−0.15^*^	−0.19^**^	0.20^**^	−0.19^**^	−0.16^*^	0.08	0.50^**^				
15. T6 Internalizing	−0.25^**^	−0.03	0.09	0.46^**^	0.17^*^	0.05	−0.06	−0.02	0.16	−0.15	−0.13	0.07	0.38^**^	0.17^*^			
16. T6 Externalizing	0.10	−0.09	0.17^*^	0.25^**^	0.11	0.22^**^	−0.14	−0.10	0.16	−0.11	−0.17	0.07	0.28^**^	0.33^**^	0.41^**^		
17. T7 Internalizing	−0.31^**^	0.04	0.18^*^	0.26^**^	0.11	0.01	0.07	0.07	0.21^*^	−0.34^**^	−0.34^**^	−0.15	0.27^**^	0.20^*^	0.59^**^	0.28^**^	
18. T7 Externalizing	0.09	−0.11	0.22^**^	0.26^**^	−0.02	0.11	−0.07	−0.13	0.18	−0.22^*^	−0.26^**^	−0.07	0.27^**^	0.40^**^	0.26^**^	0.69^**^	0.47^**^

^*^ indicates *p* < 0.05. ^**^ indicates *p* < 0.01.

### Regression analyses

#### Direct associations

In initial models, positive (β = −0.29) and threat (β = 0.23) appraisal and active (β = −0.30) but not avoidant (β = −0.14, *p* = 0.09) coping were moderate predictors of changes in internalizing symptoms across the pandemic (T7) in the expected directions, whereas neither appraisal nor coping predicted adolescent symptoms early in the pandemic (T6). Frustration significantly predicted T6 externalizing problems early in the pandemic, depending on which appraisal or coping variable was included in the regression (β’s = 0.15–0.17), but apart from that, there were no main effects of early-childhood temperament on adolescent psychopathology above the effects of other variables and covariates. Concurrent pandemic-related stress (β’s = 0.20–0.40) but not age 11/12 (T5) stress predicted T6 adolescent symptoms. Of the covariates, only previous symptoms (β’s = 0.26–0.65) were consistent predictors of psychopathology (standardized regression coefficients, standard errors, *p*-values, and 95% confidence intervals from the final models presented in Tables 3–6).

**Table 3:** Standardized regression coefficients, standard errors, and confidence intervals from regressions predicting adolescent psychopathology from temperament and active coping.

Parameter	*Est.*	*SE*	*p*	95% CI	*Est.*	*SE*	*p*	95% CI
T6 Internalizing					T6 Externalizing			
Intercept	−0.01	0.06	0.881	[−0.13, 0.11]	−0.01	0.07	0.888	[−0.14, 0.12]
Sex	−0.26	0.06	< 0.001	[−0.38, −0.13]	0.02	0.07	0.776	[−0.12, 0.16]
Income	0.07	0.07	0.299	[−0.06, 0.20]	0.05	0.08	0.496	[−0.10, 0.20]
Neg. Life Events	−0.02	0.07	0.742	[−0.16, 0.12]	0.08	0.08	0.322	[−0.08, 0.24]
Pandemic-related Stressors	0.42	0.06	< 0.001	[0.29, 0.54]	0.16	0.07	0.027	[0.02, 0.31]
T5 Psychopathol.	0.40	0.06	< 0.001	[0.28, 0.53]	0.31	0.08	< 0.001	[0.15, 0.46]
Fear	0.08	0.06	0.222	[−0.05, 0.20]	0.07	0.07	0.323	[−0.07, 0.22]
Frustration	0.03	0.07	0.678	[−0.10, 0.16]	0.12	0.08	0.131	[−0.04, 0.27]
Executive Control	0.01	0.07	0.894	[−0.13, 0.15]	−0.05	0.08	0.510	[−0.21, 0.10]
Delay	−0.02	0.07	0.817	[−0.15, 0.12]	0.03	0.08	0.697	[−0.12, 0.18]
Active Coping	0.00	0.06	0.958	[−0.12, 0.13]	−0.11	0.07	0.136	[−0.25, 0.04]
Fear × Act. Coping	−0.04	0.07	0.561	[−0.18, 0.10]	0.14	0.08	0.097	[−0.03, 0.30]
Frustration × Act. Coping	0.16	0.08	0.038	[0.01, 0.32]	0.22	0.09	0.016	[0.04, 0.41]
Exec. Control × Act. Coping	0.02	0.07	0.757	[−0.12, 0.17]	0.10	0.09	0.267	[−0.07, 0.27]
Delay × Act. Coping	−0.06	0.07	0.417	[−0.21, 0.09]	0.11	0.09	0.195	[−0.06, 0.28]
T7 Internalizing					T7 Externalizing			
Intercept	0.01	0.06	0.837	[−0.11, 0.14]	−0.03	0.06	0.613	[−0.15, 0.09]
Sex	−0.14	0.07	0.035	[−0.28, −0.01]	0.03	0.06	0.633	[−0.09, 0.16]
Income	−0.00	0.07	0.984	[−0.14, 0.14]	−0.05	0.07	0.482	[−0.18, 0.08]
Neg. Life Events	0.09	0.07	0.236	[−0.06, 0.23]	0.09	0.07	0.187	[−0.04, 0.23]
Pandemic-related Stressors	−0.07	0.08	0.362	[−0.22, 0.08]	0.07	0.07	0.339	[−0.07, 0.21]
T5 Psychopathology	0.55	0.07	< 0.001	[0.41, 0.69]	0.63	0.06	<0.001	[0.50, 0.76]
Fear	0.03	0.07	0.667	[−0.10, 0.16]	−0.10	0.06	0.135	[−0.22, 0.03]
Frustration	0.01	0.07	0.839	[−0.13, 0.16]	−0.03	0.07	0.688	[−0.16, 0.11]
Executive Control	0.07	0.07	0.343	[−0.07, 0.22]	0.02	0.07	0.824	[−0.12, 0.15]
Delay	0.12	0.07	0.113	[−0.03, 0.26]	−0.02	0.07	0.763	[−0.16, 0.12]
Active Coping	−0.30	0.07	< 0.001	[−0.44, −0.17]	−0.04	0.07	0.594	[−0.17, 0.10]
Fear × Act. Coping	0.10	0.08	0.210	[−0.06, 0.25]	−0.03	0.08	0.691	[−0.18, 0.12]
Frustration × Act. Coping	0.09	0.10	0.368	[−0.11, 0.29]	0.14	0.11	0.194	[−0.07, 0.34]
Exec. Control × Act. Coping	0.07	0.07	0.328	[−0.07, 0.22]	−0.02	0.07	0.779	[−0.17, 0.12]
Delay × Act. Coping	−0.14	0.08	0.096	[−0.30, 0.03]	−0.19	0.08	0.026	[−0.35, −0.02]

**Table 4:** Standardized regression coefficients, standard errors, and confidence intervals from regressions predicting adolescent psychopathology from temperament and avoidant coping.

Parameter	*Est.*	*SE*	*p*	95% CI	*Est.*	*SE*	*p*	95% CI
T6 Internalizing					T6 Externalizing			
Intercept	−0.01	0.06	0.859	[−0.13, 0.11]	−0.01	0.07	0.863	[−0.15, 0.12]
Sex	−0.27	0.06	< 0.001	[−0.39, −0.15]	0.03	0.07	0.671	[−0.11, 0.17]
Income	0.06	0.07	0.369	[−0.07, 0.19]	0.04	0.08	0.630	[−0.12, 0.19]
Neg. Life Events	−0.02	0.07	0.748	[−0.16, 0.12]	0.06	0.08	0.469	[−0.10, 0.22]
Pandemic-related Stressors	0.42	0.06	< 0.001	[0.30, 0.54]	0.21	0.07	0.004	[0.07, 0.35]
T5 Psychopathol.	0.39	0.06	< 0.001	[0.26, 0.52]	0.33	0.08	< 0.001	[0.17, 0.48]
Fear	0.07	0.06	0.273	[−0.06, 0.20]	0.07	0.07	0.337	[−0.07, 0.22]
Frustration	0.06	0.07	0.404	[−0.08, 0.19]	0.17	0.08	0.036	[0.01, 0.33]
Executive Control	0.02	0.07	0.734	[−0.11, 0.16]	−0.04	0.08	0.577	[−0.20, 0.11]
Delay	−0.02	0.07	0.727	[−0.16, 0.11]	−0.00	0.08	0.972	[−0.15, 0.15]
Avoidant Coping	0.00	0.07	0.945	[−0.13, 0.14]	0.06	0.08	0.495	[−0.10, 0.21]
Fear × Avo. Coping	−0.01	0.08	0.922	[−0.16, 0.14]	0.17	0.09	0.060	[−0.01, 0.36]
Frustration × Avo. Coping	−0.09	0.08	0.242	[−0.24, 0.06]	0.08	0.09	0.413	[−0.11, 0.26]
Exec. Control × Avo. Coping	0.17	0.07	0.020	[0.03, 0.31]	0.27	0.09	0.002	[0.10, 0.44]
Delay × Avo. Coping	−0.15	0.07	0.042	[−0.29, −0.00]	0.08	0.08	0.353	[−0.09, 0.25]
	T7 Internalizing				T7 Externalizing			
Intercept	−0.01	0.07	0.853	[−0.14, 0.12]	−0.04	0.06	0.530	[−0.16, 0.09]
Sex	−0.08	0.07	0.275	[−0.22, 0.06]	0.04	0.07	0.589	[−0.09, 0.16]
Income	−0.03	0.07	0.711	[−0.17, 0.12]	−0.09	0.07	0.208	[−0.22, 0.05]
Neg. Life Events	0.10	0.08	0.197	[−0.05, 0.26]	0.11	0.07	0.124	[−0.03, 0.26]
Pandemic-related Stressors	−0.06	0.08	0.455	[−0.22, 0.10]	0.06	0.07	0.402	[−0.08, 0.20]
T5 Psychopathology	0.64	0.08	< 0.001	[0.48, 0.80]	0.63	0.07	< 0.001	[0.50, 0.76]
Fear	0.09	0.07	0.204	[−0.05, 0.24]	−0.06	0.07	0.350	[−0.20, 0.07]
Frustration	−0.02	0.08	0.791	[−0.17, 0.13]	−0.04	0.07	0.557	[−0.19, 0.10]
Executive Control	0.11	0.08	0.152	[−0.04, 0.26]	0.07	0.07	0.354	[−0.07, 0.21]
Delay	0.06	0.08	0.394	[−0.09, 0.22]	−0.05	0.07	0.458	[−0.19, 0.09]
Avoidant Coping	−0.13	0.08	0.116	[−0.29, 0.03]	−0.05	0.08	0.520	[−0.22, 0.11]
Fear × Avo. Coping	0.03	0.10	0.793	[−0.18, 0.23]	−0.00	0.11	0.988	[−0.22, 0.22]
Frustration × Avo. Coping	0.25	0.11	0.024	[0.03, 0.47]	0.07	0.12	0.574	[−0.17, 0.32]
Exec. Control × Avo. Coping	−0.05	0.08	0.542	[−0.21, 0.11]	0.02	0.09	0.788	[−0.14, 0.19]
Delay × Avo. Coping	0.04	0.09	0.670	[−0.14, 0.22]	0.01	0.09	0.946	[−0.17, 0.18]

**Table 5:** Standardized regression coefficients, standard errors, and confidence intervals from regressions predicting adolescent psychopathology from temperament and positive appraisal.

Parameter	*Est.*	*SE*	*p*	95% CI	*Est.*	*SE*	*p*	95% CI
T6 Internalizing					T6 Externalizing			
Intercept	−0.01	0.06	0.894	[−0.13, 0.11]	−0.02	0.07	0.785	[−0.16, 0.12]
Sex	−0.27	0.06	< 0.001	[−0.40, −0.15]	0.03	0.07	0.677	[−0.11, 0.17]
Income	0.07	0.07	0.254	[−0.05, 0.20]	0.08	0.08	0.328	[−0.08, 0.23]
Neg. Life Events	−0.03	0.07	0.645	[−0.17, 0.11]	0.07	0.08	0.363	[−0.09, 0.24]
Pandemic-related Stressors	0.42	0.06	< 0.001	[0.29, 0.54]	0.20	0.07	0.006	[0.06, 0.34]
T5 Psychopathol.	0.40	0.07	< 0.001	[0.26, 0.52]	0.27	0.08	< 0.001	[0.12, 0.42]
Fear	0.08	0.06	0.218	[−0.05, 0.20]	0.08	0.07	0.268	[−0.06, 0.22]
Frustration	0.04	0.07	0.604	[−0.10, 0.17]	0.13	0.08	0.098	[−0.02, 0.28]
Executive Control	0.03	0.07	0.702	[−0.11, 0.16]	−0.05	0.08	0.521	[−0.21, 0.10]
Delay	−0.03	0.07	0.626	[−0.17, 0.10]	0.01	0.08	0.908	[−0.15, 0.16]
Positive Appraisal	−0.04	0.07	0.592	[−0.18, 0.10]	−0.09	0.08	0.274	[−0.24, 0.07]
Fear × Pos. Appraisal	0.00	0.07	0.951	[−0.14, 0.15]	0.09	0.08	0.266	[−0.07, 0.26]
Frustration × Pos. Appraisal	0.14	0.10	0.143	[−0.05, 0.32]	0.32	0.11	0.003	[0.11, 0.52]
Exec. Control × Pos. Appraisal	−0.01	0.08	0.849	[−0.16, 0.14]	0.01	0.09	0.945	[−0.16, 0.18]
Delay × Pos. Appraisal	−0.00	0.08	0.960	[−0.16, 0.15]	0.10	0.09	0.261	[−0.07, 0.27]
	T7 Internalizing				T7 Externalizing			
Intercept	0.01	0.06	0.876	[−0.12, 0.14]	−0.03	0.06	0.620	[−0.15, 0.09]
Sex	−0.14	0.07	0.045	[−0.28, −0.00]	0.03	0.06	0.668	[−0.10, 0.15]
Income	0.01	0.07	0.913	[−0.13, 0.14]	−0.07	0.07	0.312	[−0.20, 0.06]
Neg. Life Events	0.10	0.07	0.191	[−0.05, 0.25]	0.09	0.07	0.189	[−0.05, 0.23]
Pandemic-related Stressors	−0.04	0.07	0.596	[−0.19, 0.11]	0.06	0.07	0.398	[−0.08, 0.20]
T5 Psychopathology	0.51	0.07	< 0.001	[0.37, 0.65]	0.62	0.07	< 0.001	[0.49, 0.75]
Fear	0.07	0.07	0.323	[−0.07, 0.20]	−0.08	0.06	0.246	[−0.20, 0.05]
Frustration	0.01	0.07	0.923	[−0.13, 0.15]	−0.04	0.07	0.589	[−0.17, 0.10]
Executive Control	0.10	0.07	0.172	[−0.04, 0.24]	0.05	0.07	0.513	[−0.09, 0.18]
Delay	0.09	0.08	0.228	[−0.06, 0.24]	−0.02	0.07	0.821	[−0.16, 0.13]
Positive Appraisal	−0.28	0.07	< 0.001	[−0.42, −0.14]	−0.05	0.07	0.502	[−0.20, 0.10]
Fear × Pos. Appraisal	0.05	0.08	0.539	[−0.11, 0.20]	0.00	0.08	0.971	[−0.16, 0.17]
Frustration × Pos. Appraisal	0.18	0.12	0.132	[−0.05, 0.41]	0.12	0.14	0.418	[−0.16, 0.39]
Exec. Control × Pos. Appraisal	0.05	0.07	0.487	[−0.09, 0.20]	−0.02	0.08	0.767	[−0.17, 0.13]
Delay × Pos. Appraisal	−0.06	0.08	0.442	[−0.23, 0.10]	−0.12	0.09	0.206	[−0.29, 0.06]

**Table 6:** Standardized regression coefficients, standard errors, and confidence intervals from regressions predicting adolescent psychopathology from temperament and threat appraisal.

Parameter	*Est.*	*SE*	*p*	95% CI	*Est.*	*SE*	*p*	95% CI
	T6 Internalizing				T6 Externalizing			
Intercept	0.01	0.06	0.877	[−0.11, 0.13]	−0.02	0.07	0.810	[−0.15, 0.12]
Sex	−0.26	0.06	< 0.001	[−0.39,-0.14]	0.06	0.07	0.450	[−0.09, 0.20]
Income	0.08	0.07	0.207	[−0.05, 0.21]	0.10	0.08	0.216	[−0.06, 0.25]
Neg. Life Events	−0.04	0.07	0.585	[−0.18, 0.10]	0.07	0.08	0.392	[−0.09, 0.23]
Pandemic-related Stressors	0.42	0.06	< 0.001	[0.29, 0.54]	0.21	0.07	0.003	[0.07, 0.36]
T5 Psychopathology	0.42	0.07	< 0.001	[0.29, 0.55]	0.26	0.08	0.001	[0.11, 0.42]
Fear	0.09	0.06	0.164	[−0.04, 0.22]	0.09	0.07	0.229	[−0.06, 0.24]
Frustration	0.05	0.07	0.422	[−0.08, 0.19]	0.15	0.08	0.049	[0.00, 0.31]
Executive Control	0.03	0.07	0.673	[−0.11, 0.17]	−0.04	0.08	0.646	[−0.20, 0.12]
Delay	−0.03	0.07	0.697	[−0.16, 0.11]	0.04	0.08	0.660	[−0.12, 0.19]
Threat Appraisal	−0.03	0.07	0.732	[−0.17, 0.12]	0.09	0.09	0.316	[−0.08, 0.26]
Fear × Threat Appraisal	0.08	0.09	0.369	[−0.09, 0.25]	0.05	0.10	0.611	[−0.15, 0.26]
Frustration × Threat Appraisal	−0.08	0.09	0.350	[−0.25, 0.09]	−0.15	0.11	0.167	[−0.35, 0.06]
Exec. Control × Threat Appraisal	0.09	0.08	0.252	[−0.06, 0.25]	−0.02	0.10	0.861	[−0.21, 0.17]
Delay × Threat Appraisal	0.02	0.07	0.792	[−0.13, 0.17]	0.10	0.09	0.267	[−0.07, 0.27]
	T7 Internalizing				T7 Externalizing			
Intercept	−0.01	0.07	0.921	[−0.14, 0.12]	−0.03	0.06	0.652	[−0.16, 0.10]
Sex	−0.11	0.07	0.121	[−0.25, 0.03]	0.03	0.07	0.678	[−0.10, 0.16]
Income	−0.02	0.07	0.784	[−0.17, 0.12]	−0.08	0.07	0.291	[−0.22, 0.06]
Neg. Life Events	0.11	0.08	0.175	[−0.05, 0.26]	0.11	0.07	0.123	[−0.03, 0.26]
Pandemic-related Stressors	−0.02	0.08	0.784	[−0.18, 0.13]	0.07	0.07	0.367	[−0.08, 0.22]
T5 Psychopathology	0.56	0.07	< 0.001	[0.42, 0.70]	0.62	0.08	< 0.001	[0.47, 0.77]
Fear	0.07	0.07	0.331	[−0.07, 0.21]	−0.08	0.07	0.256	[−0.21, 0.06]
Frustration	−0.02	0.07	0.820	[−0.16, 0.13]	−0.06	0.07	0.417	[−0.20, 0.08]
Executive Control	0.13	0.08	0.075	[−0.01, 0.28]	0.07	0.07	0.354	[−0.07, 0.21]
Delay	0.04	0.08	0.566	[−0.11, 0.20]	−0.06	0.07	0.383	[−0.21, 0.08]
Threat Appraisal	0.17	0.09	0.050	[−0.00, 0.35]	0.07	0.10	0.459	[−0.12, 0.26]
Fear × Threat Appraisal	−0.00	0.11	0.980	[−0.22, 0.21]	−0.05	0.12	0.701	[−0.29, 0.19]
Frustration × Threat Appraisal	−0.06	0.13	0.631	[−0.31, 0.19]	−0.09	0.15	0.546	[−0.38, 0.20]
Exec. Control × Threat Appraisal	−0.18	0.10	0.077	[−0.37, 0.02]	0.03	0.11	0.803	[−0.19, 0.24]
Delay × Threat Appraisal	0.02	0.09	0.806	[−0.15, 0.19]	−0.04	0.09	0.708	[−0.22, 0.15]

#### Temperament moderating coping

Final models including interaction effects are reported in Tables 3–6.

##### Fear

There were no significant interactions between fear and coping or appraisal. There were only trends toward interaction effects for fear x active (β = 0.14, *p* = 0.10) and avoidant (β = 0.17, *p* = 0.06) coping predicting T6 externalizing symptoms.

##### Frustration

The interaction between frustration and active coping predicted T6 adolescent internalizing (β = 0.16, *p* = 0.038) and externalizing (β = 0.22, *p* = 0.016) symptoms. Frustration also interacted with positive appraisal to predict T6 externalizing (β = 0.32, *p* = 0.003) but not internalizing symptoms. In addition, frustration moderated the association between avoidant coping and T7 internalizing symptoms (β = 0.25, *p* = 0.024).

**Figure 2:**
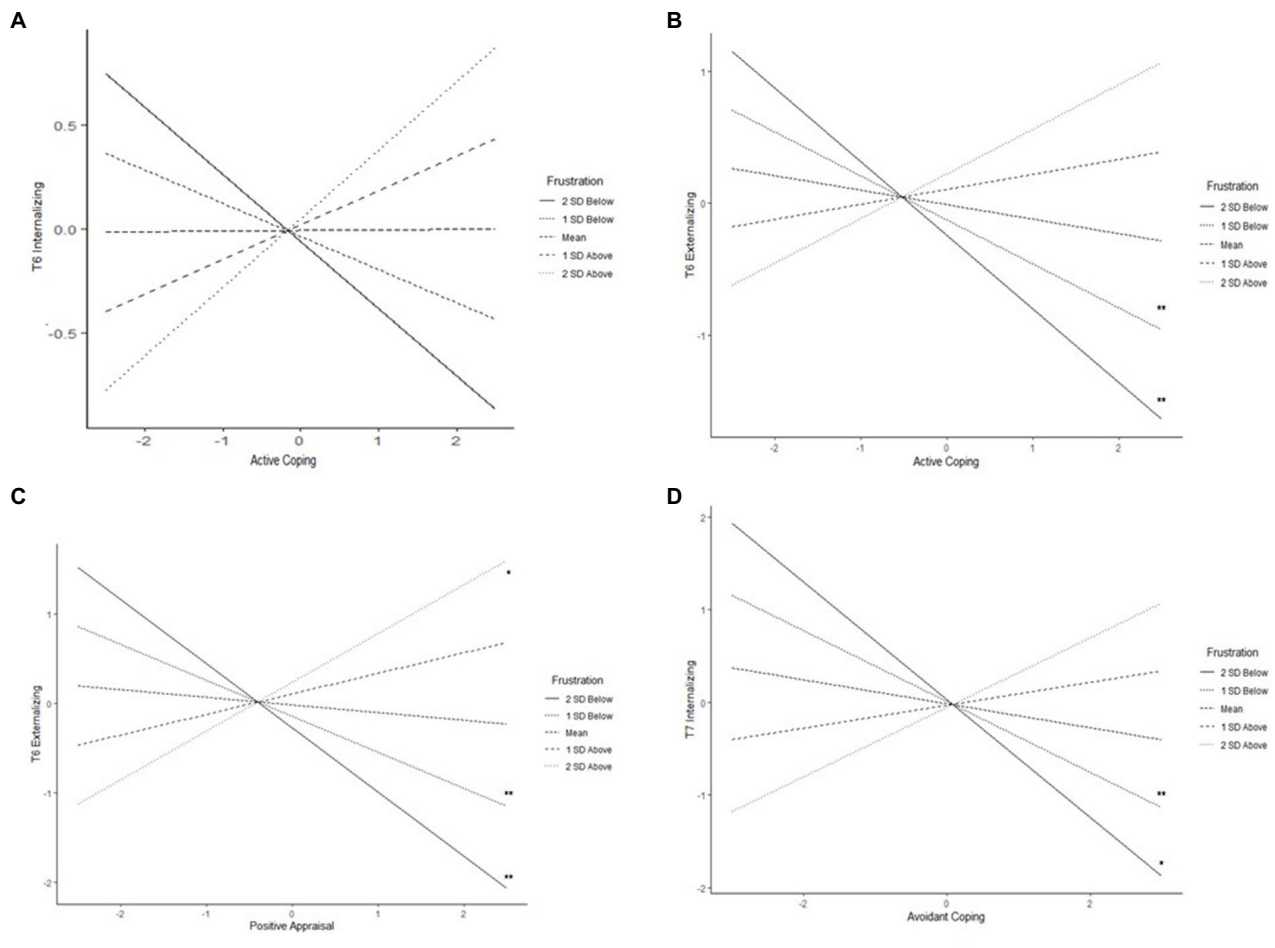
Frustration moderating the associations of **(A)** active coping with T6 internalizing, **(B)** active coping with T6 externalizing, **(C)** positive appraisal with T6 externalizing, and **(D)** avoidant coping with T7 internalizing (^*^ *p* < 0.05, ^**^ *p* < 0.01).

For T6 internalizing symptoms, although the interaction is significant, the simple slopes for frustration were not significant, indicating that slopes were significantly different from each other, but it remains unclear what levels of frustration the association between internalizing symptoms and active coping were significant. However, at lower levels of frustration, active coping was negatively related to internalizing, whereas at higher levels of frustration it was positively related (Figure 2A). For externalizing, at low levels of frustration, active coping (Figure 2B) was negatively related to externalizing symptoms whereas at mean and higher levels of frustration, active coping was not significantly associated with externalizing. Positive appraisal was negatively associated with externalizing symptoms at lower levels of frustration, but it was also associated with higher externalizing symptoms at higher levels of frustration (Figure 2C). Avoidant coping was negatively related to T7 internalizing symptoms at low levels of frustration, but unrelated to internalizing at mean and higher levels of frustration (Figure 2D).

##### Executive control

**Figure 3:**
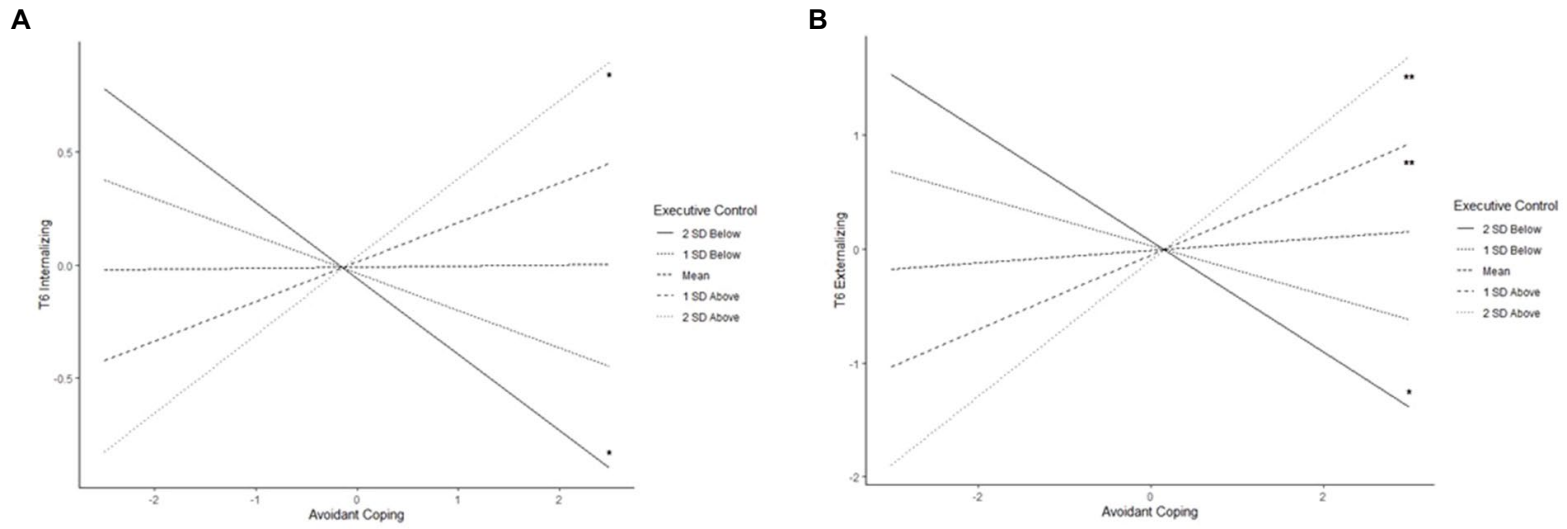
Executive control moderating the associations of **(A)** avoidant coping with T6 internalizing and **(B)** avoidant coping with T6 externalizing (^*^ *p* < 0.05, ^**^ *p* < 0.01).

Executive control moderated the association of avoidant coping with both T6 internalizing (β = 0.17, *p* = 0.020) and externalizing symptoms (β = 0.27, *p* = 0.002). Probing this interaction revealed that avoidant coping was negatively associated with internalizing and externalizing symptoms at low levels of executive control, whereas at high levels of executive control, avoidant coping was positively related to internalizing and externalizing at T6 (Figures 3A,B). Given this pattern of finding was inconsistent with our hypotheses, we examined mean levels of internalizing and externalizing at low and high levels of executive control and avoidant coping to contextualize the results. For adolescents who were low in executive control, as the level of use of avoidant coping increased, level of internalizing decreased. However, youth with lower executive control and higher avoidant coping had the highest levels of internalizing (M = 4.61, SD = 3.11) compared to others (M = 4.00 SD = 2.90). Similarly, for adolescents who were low in executive control, as use of avoidant coping increased, levels of externalizing decreased. However, youth with higher executive control and lower avoidant coping had the lowest mean levels of externalizing (M = 4.42, SD = 2.68) compared to all others (M = 5.31, SD = 2.75).

##### Delay ability

**Figure 4:**
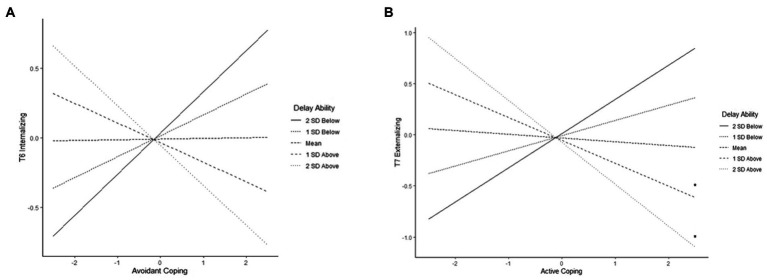
Delay ability moderating the associations of **(A)** avoidant coping with T6 internalizing and **(B)** active coping with T7 externalizing (^*^p < 0.05, ^**^p < .0.01).

Delay and avoidant coping interacted to predict T6 internalizing symptoms (β = −0.15, *p* = 0.042). Simple slopes were not significant, again indicating that the slopes were different than each other, but it is unclear at what level of delay the associations of avoidant coping with internalizing might be significant (Figure 4A). For those low in delay ability, avoidant coping was positively related to internalizing, whereas for those high in delay ability, it was negatively related to internalizing. In addition, delay ability and active coping interacted to predict T7 externalizing symptoms (β = −0.19, *p* = 0.026), and probes of simple slopes indicated that at high levels of delay ability, active coping was associated with lower T7 externalizing (Figure 4B).

## Discussion

Child temperament, early life stress, and appraisal and coping styles may serve as factors of risk and resilience in children and adolescents. Their interaction may reflect combined characterological and intentional emotion regulation efforts in contexts of stress, and the combination may be particularly relevant in understanding the development of psychopathology. In this study, we examined whether appraisal and coping styles were more or less effective in preventing symptoms of psychopathology given different temperament characteristics. We did so by examining the extent to which temperament altered the associations of appraisal and coping with changes in internalizing and externalizing symptoms in adolescents during the COVID-19 pandemic, accounting for experiences of stress. The COVID-19 pandemic presented a situation that introduced new stressors or exacerbated existing ones for many adolescents and their families, providing an opportunity to examine the prospective effects of interactions of temperament with appraisal and coping, over and above the previously existing context of stress. We found that all facets of temperament except fear moderated coping or appraisal in predicting adolescent symptoms of psychopathology. In particular, the impacts of both active and avoidant coping, as well as positive appraisal varied with temperament. However, the patterns of interactions were not all consistent with the hypothesized effects, as we discuss below. Thus, the hypothesized vulnerability model was not consistently supported.

We note that there were few significant interactions among the many tested. There were also relatively few direct effects, with direct effects of prior levels of psychopathology and pandemic-related stressors being the most consistent predictors of both initial pandemic levels of psychopathology and changes across the pandemic. Importantly, levels of psychopathology prior to the pandemic were correlated with the family’s income and experiences of stress. Taken together, the findings suggest that it is critical to account for the context of stress in understanding the potential roles of temperament, appraisal and coping in children’s psychopathology. Given this, it may be understandable that there were relatively few interaction effects and fewer direct effects of temperament, appraisal, and coping once the substantial effects of context and prior psychopathology were accounted.

We hypothesized that high fear and frustration, and low effortful control would confer risk for increased adolescent psychopathology in the context of ostensible adaptive or maladaptive appraisal and coping, while low fear and frustration and high effortful control may serve as protective factors. In partial support for this, we found that active coping and positive appraisal were related to decreases in externalizing problems at low levels of frustration. The association of active coping with internalizing was similar. However, positive appraisals were related to increase externalizing at high levels of frustration. These results indicate that frustration, positive appraisal, and active coping prior to the pandemic interacted to contribute to relative changes in psychopathology early in the pandemic. After several months of the pandemic, temperamental frustration and pre-pandemic styles of coping also predicted changes in psychopathology. At the T7 follow-up, avoidant coping was associated with lower internalizing symptoms at low frustration, and active coping was related to lower externalizing symptoms at high levels of delay. These patterns were aligned with expectations and a vulnerability model. In contrast to expectations, low executive control did not exacerbate the impact of avoidant coping, nor was high executive control protective. In fact, avoidant coping was related to decreases in internalizing and externalizing only at low levels of EC, while at high levels of executive control avoidant coping was related to increases in internalizing and externalizing symptoms, suggesting that avoidant coping was a helpful coping strategy for some children. This finding reflects that children who had a style of avoidant coping combined with low levels of executive control had the highest levels of psychopathology compared to those lower in avoidance or higher in executive control. In addition, both avoidant coping and lower executive control were related to higher levels of psychopathology prior to the pandemic, which was the most robust predictor of psychopathology in response to the pandemic.

There is ample evidence of direct effects of temperament on psychopathology, and we observed significant correlations of frustration, executive control, and delay ability with pre-pandemic levels of psychopathology. However, we did not find direct effects of early-childhood temperament on changes in adolescent symptoms, other than the association of frustration with increases in externalizing, in response to the pandemic. Rather, the findings suggest that early-childhood temperament might contribute to later psychopathology by influencing levels of psychopathology established in childhood, and it might contribute to changes in adolescent adjustment through its moderation of the effectiveness of appraisal and coping strategies employed. Few prior studies have examined temperament as a moderator of the associations of appraisal and coping with child psychopathology. However, those studies have tended to show that appraisal and coping operate differently depending on child temperament. For example, the impact of active coping on youth internalizing symptoms depended on level of negative emotionality (Sugimura et al., 2014). In another study, self-regulation altered the associations of active and avoidant coping with child anxiety (Lengua and Sandler, 1996). These findings underscore the role of individual emotionality and self-regulation in youth adjustment. Beyond direct effects, temperament contributes to psychopathology through its interplay with other risk and protective factors (Nigg, 2006), in this case, coping. Moreover, we found these effects above and beyond the impact of negative life events and pandemic-related stressors. Across all models, while pandemic-related stress was related to COVID-19 psychopathology, its impact did not subsume the effects of temperament interactions with appraisal and coping. These results suggest that while temperament predicted children’s level of psychopathology, appraisal and coping were more relevant predictors of their responses to their current context of stress.

Though both appraisal and coping were examined, coping emerged as particularly relevant to adolescents’ adjustment in response to the stressors experienced during the pandemic. Since coping is theorized to arise as a result of appraisal that may be characterized by strong negative affect, coping strategies must be responsive not only to initial appraisals, but also to thoughts and feelings that emerge in the process (Folkman and Moskowitz, 2004). This ongoing and responsive role of coping may be more sensitive to contexts, and thus may account for the significant impact seen here. Appraisal style predicted youth mental health but was less often modulated by early temperament, suggesting that specific coping behaviors may be a more important factor in managing mental health during difficult times.

### Active coping and positive appraisal

Frustration appears to play a key role in increases in the development of psychopathology, as a consistent moderator of both coping and appraisal to predict adjustment. Frustration has been theoretically and empirically associated with externalizing and social problems (Eisenberg et al., 2001; Dodge and Pettit, 2003; Muris and Ollendick, 2005; Muris et al., 2007; Nigg, 2017). This relation is theorized to emerge partly because frustrative feelings often engender aggressive behavior (Berkowitz, 1993), and frustration is theorized to emerge due to a blocked goal or reward in the activation of the behavioral activation system (BAS), theorized to underlie approach behaviors and reward sensitivity (Gray, 1982; McNaughton and Gray, 2000). In our results, active coping and positive appraisal predicted decreased externalizing symptoms only at low levels of frustration, but failed to do so in youth with higher levels of frustration, consistent with a vulnerability model. Low irritability and reactivity to blocked goals may create an ideal environment for appraisal and coping strategies characterized by engagement and anticipation of success. On the other hand, evidence suggests that high sensitivity to reward and frustration is more related to the use of disengagement strategies (Melegari et al., 2021). High frustration was indeed a vulnerability in that it interacted with positive appraisal to predict higher levels of externalizing symptoms. Positive appraisals reflect expectations for goal attainment or a positive outcome and sufficient resources to achieve that (Lengua and Long, 2002). But as high frustration is associated with proneness to anger, irritability, and sensitivity to blocked goals, barriers to acting on positive appraisals may result in frustrated attempts at resolution or emotion regulation (Kuppens and Van Mechelen, 2007). Moreover, in this case, positive appraisals may indicate a potential undervaluation of challenge or overevaluation of effective adequate resources. Other research has found that stress appraisals underestimating challenge were associated with increased externalizing symptoms in adolescence (Conway et al., 2016).

Delay ability is thought to stem from reward-sensitive systems, and to also reflect sensitivity to blocked goals. Delay ability moderated the impact of coping on adolescent psychopathology. For those who were higher in delay ability, active coping was associated with lower levels of externalizing, whereas those low in delay ability trended toward higher externalizing at higher levels of active coping, again, consistent with a vulnerability model. This suggests that delay ability supported more effective use of active coping, and being low in delay ability rendered active coping ineffective. The motivational and regulatory skills in emotionally heightened contexts that underlie delay ability may aid in navigating affect (Mischel et al., 2011), particularly in the context of situationally appropriate coping strategies.

### Avoidant coping

The pattern of interactions of temperament with avoidance were not in the hypothesized direction, and were not consistent with a vulnerability model. Controllability is an important factor to consider in the context of coping. Less controllability has been associated with likelihood of youth engaging in more avoidant coping styles (Zimmer-Gembeck et al., 2016), and avoidant coping styles have been associated with better outcomes for children who faced less controllable, acute stressors (Aldridge and Roesch, 2007). Proactive avoidance, identifying, assessing, and taking steps to minimize or avoid threat impact (LeDoux and Gorman, 2001; Hofmann and Hay, 2018), may also be a useful framework for considering how adolescents are engaging in avoidance during the time of the pandemic. A number of studies early in the pandemic found avoidant coping to be positively related to distress among adults (Dawson and Golijani-Moghaddam, 2020; Rettie and Daniels, 2020; Wang et al., 2020). Other research has found that avoidant/disengagement coping was similarly associated with lower general distress (Hsieh et al., 2021) or not at all related to mood (Wang et al., 2021) in adolescents. As these interactions predicted T7 internalizing several months into the pandemic, it suggests that avoidance might have been particularly relevant at a time in which teens identified many stressors as beyond their control and took steps to avoid their impact.

In the case of internalizing symptoms, avoidant coping was related to lower problems for those with low executive control and low frustration. Previous research has found avoidant style coping to be related to lower externalizing among young boys (Blair et al., 2004). On the other hand, executive control has been implicated as a protective factor in the development of psychopathology. Specifically, low executive control has been linked to higher internalizing and externalizing problems (Razza et al., 2010; Nigg, 2017). This association is thought to be partially accounted for by an inability to regulate attention to stimuli evoking negative emotion, as well as difficulty executing cognitive coping strategies (e.g., cognitive reappraisal) and regulating appropriate behavioral responses to dysphoria (Nigg, 2017). In this context, avoidant coping may result in less distress and fewer adjustment problems as alternative coping strategies, particularly those that might require attentional flexibility or shifting such as cognitive reappraisal, are less available or less effective for youth with lower executive control. In addition, avoidant coping may avert exacerbation of symptoms through experiences of failure in executing active strategies, which require more cognitive control and planning. Avoidant coping might be a compensatory emotion regulation strategy that is effective in reducing distress when someone is temperamentally more prone to distress due to high frustration or low effortful control. However, as noted above, this pattern of interaction also reflected that lower executive control and a style of avoidant coping were each related to higher levels of psychopathology prior to the pandemic, and that youth both high in avoidance and low in effortful control had the highest, albeit decreasing, levels of psychopathology during the pandemic, pointing to a potential ceiling effect.

Frustration has been associated with internalizing symptoms as well, specifically depression (Oldehinkel et al., 2004; Nigg, 2006). While high frustration may be related to increased dysphoria when goals are blocked, low frustration may also be related to lower motivation and approach of goal receipt. Avoidance may be more tenable in the context of low approach related to low frustration, and avoidance may also mitigate increased dysphoria from unmotivated or unsuccessful attempts at active coping. Moreover, the observed frustration used in this study may obscure other aspects of frustrative temperament. Zalewski et al. (2011) identified different patterns of frustrative profiles in children comprised observed, physiological indicators (heart rate), and self-reported frustration. The profile of moderate to low observed frustration but higher physiological and self-reported measures was positively associated with depressive symptoms (Zalewski et al., 2011). It is possible that avoidant strategies ameliorate the mood impact of these other frustrative characteristics. Overall, our findings suggest that low levels of frustration may indeed be protective across coping and appraisal strategies.

While this study’s use of behavioral measures provided more objective indication of individual temperament, these measures might not capture patterns of regulation and reactivity across time and situations (e.g., Hubbard et al., 2010). This may explain the lack of direct or interactive effects with fear and adjustment. However, observations across four assessments that spanned two and half years were aggregated, capturing the consistency of the observations across time. In addition, the use of early-childhood observational measures of temperament reduced concerns about the potential mutual influences of temperament with stress, appraisal, and coping shaping temperament over time. The assessments occurred prior to assessments of appraisal, coping, stress, and psychopathology. Given prior evidence that has shown potential associations among these variables over time (e.g., Thompson et al., 2014, 2016), the early-childhood assessments captured children’s temperament characteristics prior to substantial collinearity. Nonetheless, our results suggest that early life negative emotionality and effortful control may interact with coping strategies to impact the development of internalizing and externalizing symptoms in adolescence.

### Strengths and Limitations

This study had several strengths. A key strength of this study was its developmental framework in which we were able to leverage longitudinal data from multiple-reporters, observational data, and an economically diverse sample across seven timepoints during childhood and adolescence. There were limitations of the study as well. While it was economically diverse, the sample used in this study was less diverse than the original sample, limiting our ability better generalize the findings. Our measure of coping and appraisal asked individuals to independently generate problems for coping and appraisal which may have led to differential responses. We also used broad categories of coping rather than narrowing in on specific strategies, limiting the specificity with which our results can speak to interventions.

### Future directions and implications

Future directions in this work include examining both more specific and momentary reports (rather than global self-report) of coping strategy use. Physiological measures of regulation would also deepen our understanding of these associations. Facets of temperament are known to interact (e.g., Muris et al., 2007; Halvorson et al., 2022), thus three-way interactions may help probe emotionality and regulatory transactions with appraisal and coping. Additional considerations, such as the differential impacts of parent-level factors such as parental mental health and self-regulation, may be fruitful additions to this work. Parental self-regulation, emotionality, and mental health may all play a role in youth coping and outcomes in contexts of stress.

The results of this study underscore the role of individual emotionality and self-regulation in youth adjustment. Early individual differences in negative emotionality and self-regulation continue to contribute to psychopathology into adolescence by altering the effectiveness of coping efforts. These effects were observed over and above the effects of the context of stress, emphasizing the contribution of temperament to youth stress responses and adjustment. These findings generally suggest that equipping youth with active coping skills may serve to reduce negative mental health outcomes. Indeed, a recent meta-analysis (Eadeh et al., 2021) reported that programs to improve adolescent emotion regulation were generally effective for both clinical and community samples by either increasing active or decreasing avoidant strategies. Although this effect did not differ based on sex or age, important factors like temperament were not explored as moderators. Our results suggest that, for some youth, particularly those high in frustration and low in executive control, additional or alternative emotion regulation strategies might be needed to support effective coping. Interventions might incorporate compensatory strategies or training to enhance inhibitory control (e.g., Rossignoli-Palomeque et al., 2019) or mindfulness practices (e.g., Long et al., 2021) to complement cognitive-behavioral coping strategies. Consideration of individual temperament differences in the delivery of coping enhancement or clinical intervention can support better emotion regulation and mental health.

## Data availability statement

The original contributions presented in the study are included in the article/supplementary material, further inquiries can be directed to LL, liliana@uw.edu.

## Ethics statement

The studies involving human participants were reviewed and approved by the University of Washington Institutional Review Board and Harvard University Institutional Review Board. Written informed consent to participate in this study was provided by the participants’ legal guardian/next of kin.

## Author contributions

LL, LS, and MS contributed to conception and design of the study. LL, KM, MR, AR, and AM contributed to conception and design of the parent studies from which the data were drawn. KP, MZ, MR, AR, SK, and MM were responsible for managing data collection and organization. MS and LL performed the statistical analysis. MS wrote the initial draft of the manuscript. KP, LS, LL, and MS wrote sections of the manuscript. All authors contributed to the article and approved the submitted version.

## Funding

This work was supported by the National Institute of Child Health and Human Development (R01 HD054465 to LL), National Institute of Mental Health (R01 MH106482 to KM), and the Bezos Family Foundation (to AM).

## Conflict of interest

The authors declare that the research was conducted in the absence of any commercial or financial relationships that could be construed as a potential conflict of interest.

## Publisher’s note

All claims expressed in this article are solely those of the authors and do not necessarily represent those of their affiliated organizations, or those of the publisher, the editors and the reviewers. Any product that may be evaluated in this article, or claim that may be made by its manufacturer, is not guaranteed or endorsed by the publisher.
